# Safety and efficacy of endoscopic retrograde cholangiopancreatography in previously treated liver cancer patients: a survival analysis

**DOI:** 10.3389/fonc.2023.1231884

**Published:** 2023-07-19

**Authors:** Hong-Yu Li, Lijun Jia, Wujun Du, Xiao-Rong Huang

**Affiliations:** ^1^ Department of Gastroenterology, The People's Hospital of Changxing Country, Zhejiang, China; ^2^ Department of Anesthiology and Perioperative Medicine, The First Affiliated Hospital of Zhengzhou University, Zhengzhou, China; ^3^ Department of Emergency Surgery, General Hospital of Ningxia Medical University, Yinchuan, China

**Keywords:** liver cancer, liver cancer treatment, biliary complications, patient’s survival rate, endoscopic retrograde cholangiopancreatography

## Abstract

**Background and Aim:**

The prognosis and medication response for liver malignancies are both dismal and highly heterogeneous. For this diverse malignancy, multimodality therapies such as drugs, surgical management, and/or l+iver transplantation are available. Biliary complications remain a major problem after liver cancer treatment especially in those patients who undergo liver transplantation for their end stage liver disease. Although, most biliary complications can be successfully managed with endoscopic retrograde cholangiopancreatography. However, biliary complications still considered an important factor influencing long-term results in liver cancer treatment patients. The aim of this study was to evaluate the effect of biliary complications on the overall patient’s survival rate after the endoscopic retrograde cholangiopancreatography.

**Patients and Methods:**

We retrospectively analyzed data of consecutive patients who were treated for liver cancer at our tertiary care hospital from January 2015 to July 2020. We focused on the biliary complications and procedural data, including post-endoscopic retrograde cholangiopancreatography complications, survival rate, and complementary or alternative treatments to endoscopic retrograde cholangiopancreatography.

**Results:**

We identified 967 cases (mean age 49; range 11-75), 84% men. During the mean follow up of 25 months (range 1 to 66 months), 102 patients developed biliary complications; 68/102 underwent 141 therapeutics endoscopic retrograde cholangiopancreatography procedures. The rest 34/102 patients were managed with percutaneous transhepatic cholangiography, conservative management, and/or surgery. Post- endoscopic retrograde cholangiopancreatography complications occurred in 79.4%, including anastomotic strictures in 25, non-anastomotic strictures in 5, stones in 5, cholangitis in 4, post-sphinctretomy bleeding in 3, pancreatitis in 2, and bile leakage in 1 patient. Seven (13.0%) patients died after ERCP due to multiple organ dysfunction syndrome. Although the survival rate of patients who underwent ERCP and those without ERCP was similar, patients with biliary complications fared significant worse.

**Conclusion:**

Although endoscopic retrograde cholangiopancreatography is useful for the management of post liver cancer treatment biliary complications; the need for multiple rounds of endoscopic retrograde cholangiopancreatography and even post endoscopic retrograde cholangiopancreatography complications is relatively high, and often results in increased mortality. However, the survival following endoscopic or surgical therapy in liver cancer treatment patients is similar.

## Introduction

Over the past few decades, liver cancer incidence has been rising, and since 2007, liver cancer-related mortality has been increasing by more than 2% annually (1). Wide range of therapeutic options are available for patients with liver cancer, such as liver transplantation, surgical resection, percutaneous ablation, radiation, transarterial, and systemic therapy. As a result, therapeutic decision-making requires the involvement of a multidisciplinary team that continuously modifies the specific treatment plan in light of the patient’s tumor stage, liver function, and performance status (2). The most frequently occurred complications in liver cancer treatment (especially liver transplantation) patients are biliary tract complications which accounts for 5% to 35% of overall complications after liver cancer treatment. The biliary tract complications in liver cancer treatment patients can result in significant morbidity (3–7).

Endoscopic retrograde cholangiopancreatography (ERPC) procedure is used for the diagnosis and treatment of pancreatic and hepatobiliary diseases. However, complications following ERCP require specific attention and clinical practice guideline especially when ERCP is performed in liver cancer treatment patients owing to their biliary complications (8–10). If patients with liver cancer experience biliary problems following a successful liver transplant, ERCP is regarded as the gold standard treatment (11–13). Advantages of ERCP include rapid confirmation of the diagnosis and immediate therapy making percutaneous or surgical correction seldom necessary after liver transplantation. Although several studies have shown that ERCP is relatively safe and effective in the management of post liver cancer treatment biliary complications (14–16). However, complications after the ERCP itself remain a major concern in liver cancer treatment patients which is believed to have significant impact on the overall patient survival rate. To date, there is only limited data available regarding the effects of ERCP complications in liver cancer treatment patients especially liver transplantation and their effect on patient survival.

The aim of this study was to analyze our experience and results of ERCP in liver cancer treatment patients particularly who underwent liver transplantation for third end stage liver disease. In addition, we also discussed the complications and adverse events occurred after the ERCP in these patients, and their possible impact on patient’s overall survival rate.

## Materials and methods

### Patients and data collection

We retrospectively reviewed consecutive patients who had undergone liver transplantation and subsequently underwent ERCP or other interventions for biliary complications at our tertiary care hospital between January 2015 and July 2020. Interventions other than ERCP were included percutaneous transhepatic cholangiography (PTCD), biliary-enteric anastomosis, and other surgical interventions. A few patients did not undergo any intervention for their biliary complications and were managed conservatively.

The study protocol was approved from the Institutional Review Board of our tertiary care hospital. We focused on biliary complications and procedural data, including post-endoscopic retrograde cholangiopancreatography complications, survival rate, and complementary or alternative treatments to endoscopic retrograde cholangiopancreatography.

### Description of the procedures

Under general anesthesia, all the ERCP procedures were performed by the senior endoscopists with a minimum experience of 1000 ERCPs. Depending on the radiologic findings and the patient’s characteristics, the gastroenterologist/endoscopist decided on the type of treatment (dilation and/or stent placement). The indications for ERCP included stenosis, leaks, cholestasis, choledocolithiasis and stent placement/removal. If ERCP was not successful, alternative or complementary treatment percutaneous transhepatic cholangiography (PTCD) was used.

ERCP success was defined as ‘when a biliary complication was successfully managed solely by the ERCP procedure and no other intervention was required’. ERCP failure was defined as ‘when the manifestations of biliary complication remained or recurred after the ERCP procedure, and alternative treatment was required such as PTCD, surgery or both’.

### Statistical analysis

IBM SPSS Statistics version 25.0 was used to conduct the statistical analysis. Results were presented using descriptive statistics; categorical variables were given as percentages (%) and continuous variables were reported as means with a standard deviation (SD). In order to compare the variables of interest, a chi-square test was utilized. Additionally, a Kaplan-Meier survival analysis was performed. Statistical significance was defined as a p-value 0.05.

## Results

We included 967 patients (age, mean ± SD 49 ± 10 years; range 11-75), 84% men who underwent liver therapy (transplantation and/or other surgical interventions) for their end stage liver diseases. The baseline characteristics of the included cohort are summarized in **Table 1**. Over a mean follow up of 25 months (range 1 to 66 months), 102 (10.5%) patients developed biliary complications. The most common biliary complications observed was anastomotic strictures in 70 (68.6%), bile duct stones associated with stricture in 10 (9.8%), non-anastomotic strictures in 9 (8.8%), bile leaks 7 (6.9%), bile duct stones in 4 (3.9%), cholangitis in 1 (1.0%) and other in 1 (1.0%).

**Table 1:** Baseline characteristics of all the included patients before the procedure.

Characteristics	Patients developed complications	Patients without complications	*P* value
	n = 102	n = 865	
**Age (years) (IQR)**	**48 (11-70)**	**49 (14-75)**	**0.188**
**Male gender (%)**	**82 (80.4)**	**729 (84.3)**	**0.378**
**Female gender (%)**	**20 (19.6)**	**136 (15.7)**	
Indication for liver therapy (%), (n=967)			
**Hepatocellular carcinoma (HCC)**	**357 (36.9)**		**0.989**
**Hepatitis B cirrhosis**	**337 (34.9)**		
**Other**	**82 (8.5)**		
**Alcoholic cirrhosis**	**57 (5.9)**		
**Autoimmune diseases (AIH, PBC, PSC)**	**47 (4.9)**		
**Acute liver failure**	**38 (3.9)**		
**Hepatitis C cirrhosis**	**17 (1.8)**		
**Wilson disease**	**9 (0.9)**		
**Hepatitis C cirrhosis and Hepatitis B cirrhosis**	**8 (0.8)**		
**Hilar cholangiocarcinoma**	**8 (0.8)**		
**Cholangiocellular carcinoma**	**6 (0.6)**		
**Amyloid polyneuropathy**	**1 (0.1)**		

AIH, autoimmune hepatitis; ERCP, endoscopic retrograde cholangiopancreatography; PBC, primary biliary cholangitis; PSC, primary sclerosing cholangitis.

Overall, 68 patients underwent ERCP, and 141 therapeutic ERCP’s (mean 2.1 ± 1 ERCPs per patient) were performed in patients with biliary complications. The mean number of ERCPs per patients was 2.2 ± 1.5 for anastomotic strictures, 2.0 ± 1.2 for non-anastomotic strictures, 1.0 ± 0.0 times for bile duct stones, 1.9 ± 1.2 times for bile duct stones associated with strictures, and 2.0 ± 1.1 times for biliary leaks. Post-ERCP complications developed in 54/68 (79.4%) of patients; anastomotic strictures in 25 (46.3%), non-anastomotic strictures in five (9.3%), stones in five (9.3%), cholangitis in four (7.4%), post-sphinctretomy bleeding in three (5.6%), pancreatitis in two (3.7%), bile leakage in one (1.9%). Seven patients (13.0%) died after ERCP due to multiple organ dysfunction syndromes. Six patients failed ERCP and later three underwent biliary-enteric anastomosis, two were treated conservatively and one underwent PTCD. Two patients underwent re-transplantation after a successful ERCP due to elevated liver enzymes.

Seven patients underwent PTCD for the management of their biliary complications and three required surgical intervention. Twenty-five patients did not undergo any intervention and were managed conservatively.

**Figure 1:**
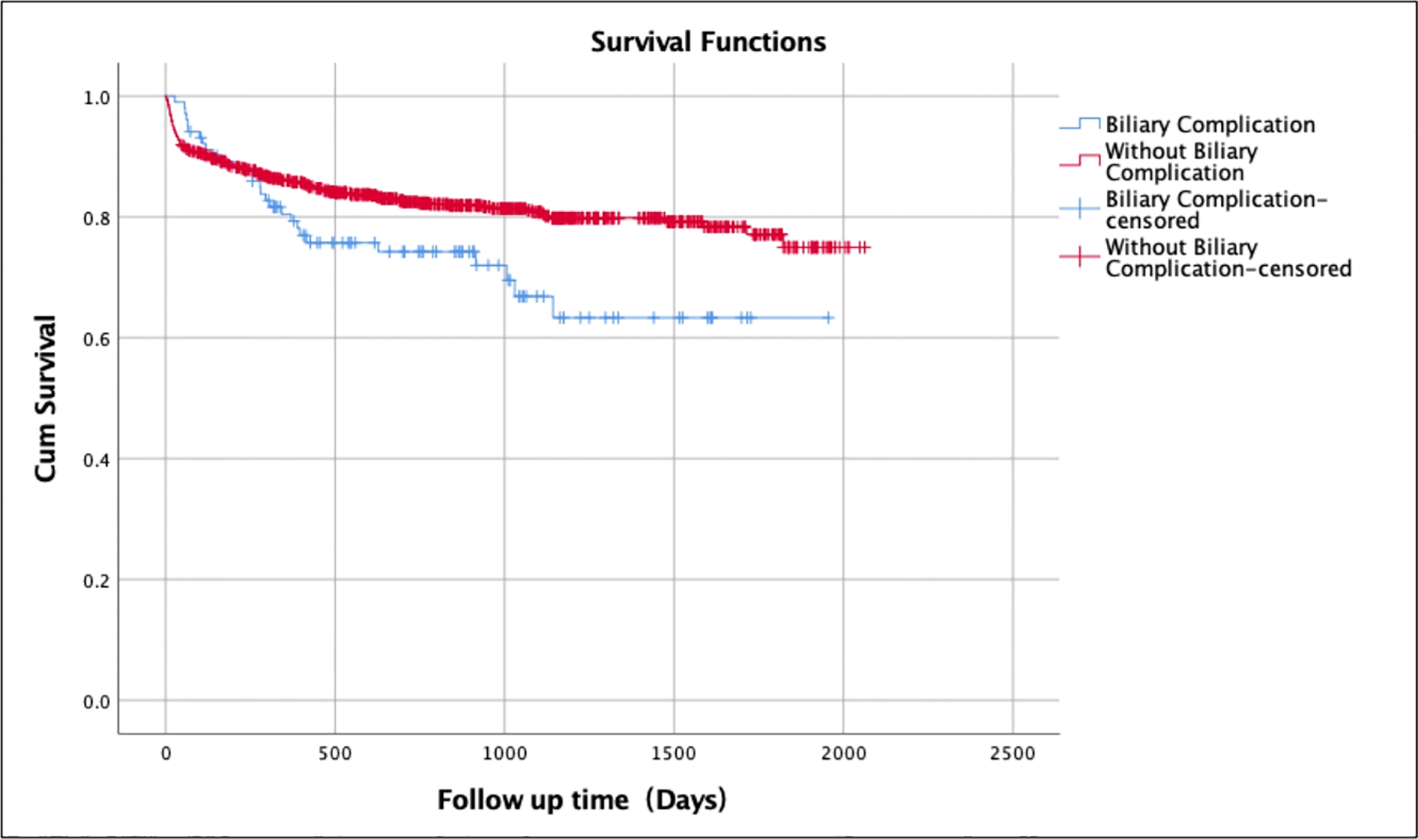
Kaplan-Meier survival analysis for patients without biliary complications vs. with biliary complications after liver transplantation.

**Figure 2:**
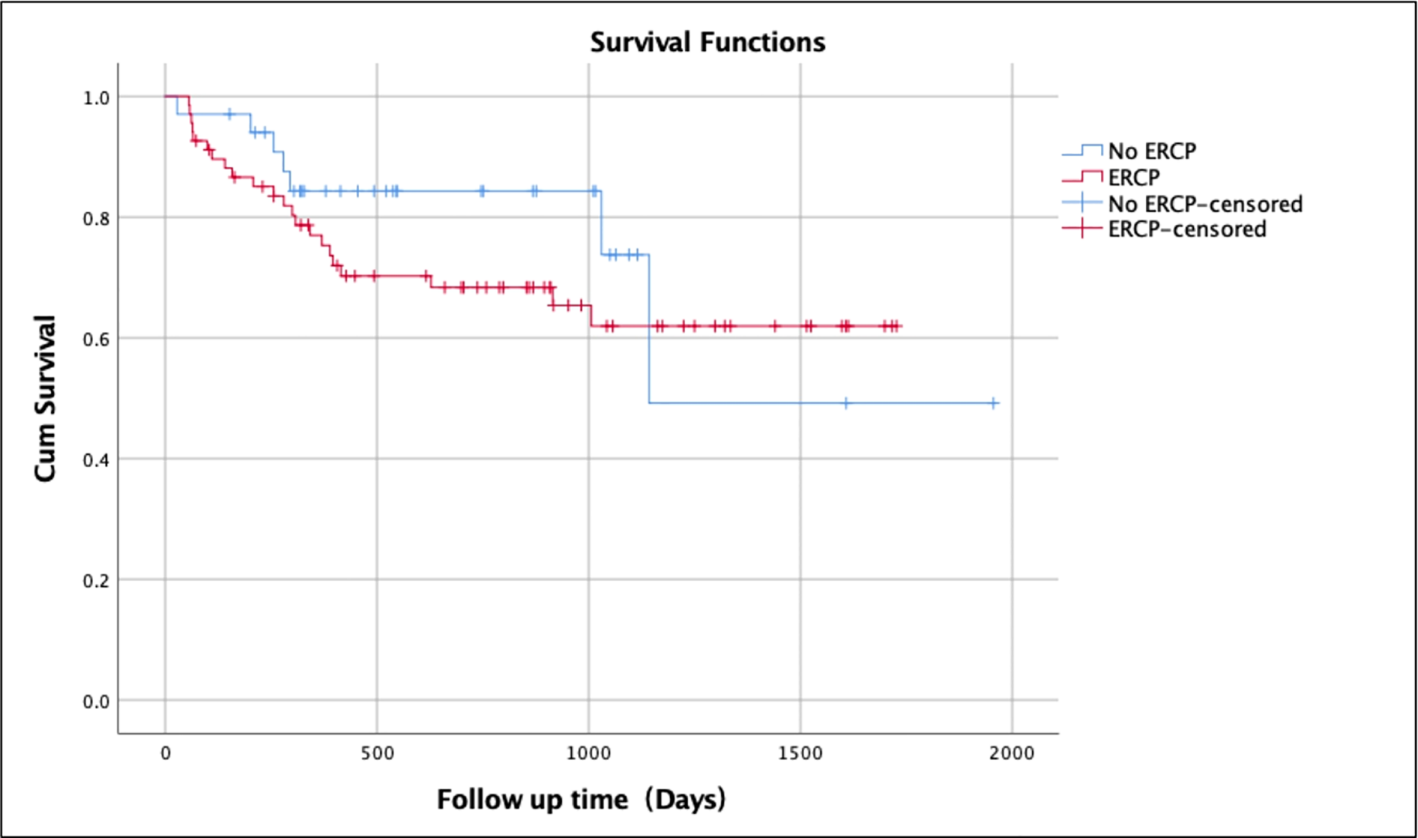
Kaplan-Meier survival analysis in ERCP patients and non-ERCP patients.

The overall survival rate over a mean follow-up duration of 25 months for patients without any biliary complications was 82.1% (mean ± SD 25.6 ± 17.4 months; range 1-66 months) (**Table 2**). The survival rate of patients with biliary complications but without ERCP was 79.4% (mean ± SD 21.1 ± 14.2 months; range 1-63 months) compared to 67.6% (mean ± SD 23.2 ± 16.3 months; range 2-56 months) of those who underwent ERCP (**Figure 1**). Patients without biliary complications had better overall survival compared to patients who had biliary complications. There was no difference of survival in post-transplant patients with biliary complications who underwent ERCP and those treated without ERCP (**Figure 2**).

**Table 2:** Survival rate of patients who developed biliary complications and underwent ERCP vs without ERCP.

Overall patients who developed biliary complication (n = 102)				
Characteristics	Patients who did not undergo ERCP procedure (n = 34)		Patients who undergo ERCP procedure (n = 68)	
	Alive	Death	Alive	Death
n (%)	27 (79.4%)	7 (20.6%)	46 (67.6%)	22 (32.4%)
Mean	651.6		722.0	
SD	441.8		506.2	
Range (days)	28-1956		56-1727	

## Discussion

The fifth most common cause of death worldwide and in the United States is liver cancer (17). The majority of patients with liver cancer receive advanced stage diagnoses, which greatly worsens the disease’s prognosis (18). The most prevalent type of liver cancer (>90%) and the main reason for cancer-related fatalities in China, the United States, and other developed countries are hepatocellular cancers (19, 20). Chronic liver disease and cirrhosis caused by the hepatitis B and/or C viruses, alcoholic liver disease, non-alcoholic fatty liver disease (NAFLD), diabetes, and obesity are the main risk factors for the development of hepatocellular carcinoma (21–23). In the Western World and Asia, chronic alcohol intake (the consumption of 40–60 g of alcohol/day), is considered as the key risk factor for the development of hepatocellular cancer (24, 25).

Chemotherapy and immunotherapy are the two most commonly used therapies for hepatocellular cancer. However, liver transplantation remains the most favorable option for the end stage liver disease (17, 26). Biliary tract complications are an important cause of morbidity and mortality after liver cancer treatment (liver transplantation) with an estimated incidence of 10% to 30% and a mortality rate of up to 10% (27, 28). Biliary complications after liver transplantation may be related to factors such as hepatic artery thrombosis or stenosis, ischemia reperfusion injury, immunologic injury, infections, and technical issues which include imperfect anastomosis and T-tube-related complications (29, 30). Most of the common biliary complications after liver transplantation are recognized clinically. Despite knowledge of the risk factors or biliary complications and improvement in both medical and surgical management, biliary complications are still considered one of the most important issues in the management of liver transplant patients. The majority of biliary complications after liver transplantation are now being managed endoscopically rather than surgically. If ERCP is unsuccessful, a percutaneous intervention or surgery is often the preferred second option (11, 31). In this study, we describe the biliary complication incidence after liver transplantation, post-ERCP complications incidence, and patient survival rate after liver transplantation in those with biliary complications vs. without biliary complications and ERCP patients and non-ERCP patients.

In our study biliary complications occurred in 10.6% patients which is consistent with previous studies. The most common complications described in previous studies are bile leaks which ranges from 2%-25% after liver transplantation (32, 33). The published literature reports that the incidence of biliary strictures ranges from 2% to 15% after deceased donor liver transplantation and 28%-32% after living donor liver transplantation (29, 34). In one study, anastomosis strictures were the most common accounting for 70% of all strictures. The reported incidence of non-anastomosis strictures differs greatly between different studies, ranging from 1% to 19%. Overall 63% of our patient sample developed strictures; of which 46% were anastomotic strictures. In most studies the incidence of biliary stones also remains a major concern after liver transplantation with the reported incidence ranging from 3.3 to 12.3% (35). Post-transplant calculi and sludge formation are most likely the result of mechanical obstruction, mainly strictures, bacterial infection, ischemia, biliary reflux, and biliary mucosal inflammation.

Biliary complications were successfully managed by ERCP without any complications in 20% of the patients who underwent ERCP in our study; 80% (54/68) developed Post-ERCP complications. The rate of complications in our study was high compared with previous studies. The type of stent might be one of the factors affecting the post-ERCP complications. At our center, single plastic stent was used in all the patients whereas several recent reported studies described the superior efficacy of multiple plastic stents or metallic stents compared to a single plastic stent. The highest clinical success rates were observed with temporary simultaneous placement of multiple plastic stents (94%), followed by placement of uncovered self-expandable metallic stents (80%), and by placement of a single plastic stent (60%). On the contrary, uncovered self-expandable metallic stents were associated with the highest complication rates (40%) compared with a single plastic stents (36%) and multiple plastic stents (20%) (36). Placing a totally covered self-expandable metal stent across the stenosis is another endoscopic technique that has been discussed. The procedure has been demonstrated to be safe, however a significant migration rate and stricture recurrence during a 5-year follow-up period have been recorded (37). Another reason of the higher complications rate in our study may be other factors such as rejection due to immunosuppressant or misdiagnosis of the complications due to the liver enzyme dysfunction. In that case, based on our experience we suggest that a biopsy should be done to determine the function of the liver on an annual basis. However, in current study, biopsy was not performed to determines the liver function which could be a future research direction.

A recent study reviewed 25050 liver transplants and the patient survival was the most commonly evaluated outcome for transplant success which ranges from 79.5% to 84.6% during the first year and 65% to 79.1% at 5 years after transplantation (38, 39). In our study, overall survival rate of patients without any biliary complications was 82.1% over a follow-up duration of 25 months. The survival rate of patients with biliary complications but without ERCP was 79.4% whereas those who underwent ERCP had 67.6% survival. Patients without biliary complications had better overall survival rate compared to patients who had biliary complications. No difference of survival in patients who underwent ERCP and those without ERCP.

In conclusion, although ERCP is the first choice, it might not be the best option for many biliary complications especially in previously treated patients for liver cancer (e.g., patients after liver transplantation). Hepatojejunonectomy/hepato-jejuno anastomosis and other interventions should be studied along with ERCP for comparative safety. As it is understood that retrospective studies have some limitations and the results can be reliable 100%. This trigger the needs for further prospective randomized studies to confirm the results shown in our studies and to effectively identify the liver transplantation recipients at risk of such complications.

## Data availability statement

The raw data supporting the conclusions of this article will be made available by the authors, without undue reservation.

## Ethics statement

The studies involving human participants were reviewed and approved by General Hospital of Ningxia Medical University. The patients/participants provided their written informed consent to participate in this study.

## Author contributions

Study concept, H-YL, LJ, and XH. Manuscript drafting, WD, LJ, H-YL, and XH. Collection and analysis of data, H-YL. Revision of the manuscript, H-YL, LJ, and XH. All authors contributed to the article and approved the submitted version.
